# Transitional, fused and/or supernumerary vertebrae in the lumbosacrocaudal region of the spine – A reality in many domestic mammals

**DOI:** 10.17221/102/2023-VETMED

**Published:** 2024-05-27

**Authors:** Costica Toader Covasa

**Affiliations:** Department of Preclinics, Faculty of Veterinary Medicine, University of Life Sciences, Iasi, Romania

**Keywords:** caudalisation, domestic species, lumbarisation, sacralisation, spine morphology

## Abstract

The aim of this study was to identify the presence of transitional lumbosacral and sacrocaudal vertebrae in domestic mammals, to realise a comparative analysis of the localisation and conformation of this abnormal condition. The research included the following species: cattle – 29 specimens, sheep – 32 specimens, horse – 31 specimens, pig – 26 specimens, rabbit – 33 specimens, dog – 89 specimens and cat – 57 specimens. The spine of the animals was analysed post-mortem or radiologically. The investigations revealed the presence of transitional vertebrae as follows: in cattle – 3 cases (8.7%), all being about the lumbarisation of the first sacral vertebra (S1); in sheep – 3 cases (9.37%), two lumbarisation cases of S1 and one caudalisation of S4 (the last sacral vertebra); in horses – 4 cases (12.9%), all about the sacralisation of Cd1 (first caudal vertebra); in pigs – 3 cases (11.53%), two lumbarisation cases of S1 and one sacralisation of Cd1; in rabbits – 3 cases (9.09%), a lumbar supernumerary vertebra (L8) and two cases of caudalisation of S4; in dogs – 4 cases (4.49%), a lumbar supernumerary vertebra (L8) and 3 cases of sacralisation of the last lumbar vertebra (L7 or L8); in cats – 3 cases (5.26%), two sacralisation cases of the last lumbar vertebra and one case of caudalisation of the last sacral vertebra (S3). A strong lumbarisation process was observed in ruminants (especially in cattle), then in pigs, the sacralisation being prevalent in carnivores. The sacrocaudal transitional vertebra was predominant in horses. No evident influence of the sex and age of the animals was observed.

Knowledge of normal spinal morphology and the incidence of vertebral anomalies is important for the distinction of pathologic changes from functional anatomic variations (Haussler et al. 1997). Vertebral congenital anomalies have been defined as defects in the normal conformation of the spine (Fluckiger et al. 2009; Westworth and Sturges 2010; Gutierrez-Quintana et al. 2014; Proks et al. 2018). Among these, the occurrence of transitional vertebrae is one of the most common anatomical abnormalities of the spine. Transitional vertebrae are considered congenital anomalies, which occur in various species of animals and in humans (Fluckiger et al. 2009; Kumar et al. 2023) and their presence can be clinically significant (Bahadir Ulger and Illeez 2020). A hereditary predisposition to transitional vertebra formation is supposed to exist (Morgan et al. 1999; Wigger et al. 2009; Fialova et al. 2014; Gluding et al. 2021). There are debates if the acquired fusion of two adjacent vertebrae can be considered as transitional vertebra (Haussler et al. 1997). Transitional vertebrae can appear in any junction of the spine presenting common characteristics of both adjacent vertebral segments. The lumbosacral transitional vertebra (LTV) is considered to be the result of an incomplete homeotic transformation of a lumbar vertebra into a sacral vertebra (sacralisation) or a sacral vertebra into a lumbar vertebra (lumbarisation) (Galis et al. 2014). The prevalence of sacralisation is higher than lumbarisation (Deppa and John 2014; Abutaher and Avinash 2019). LTV is described as a congenital anomaly which could have some effect on the spinal biomechanics and consequently could affect degenerative processes in adjacent vertebrae in mammals (Galis et al. 2014). Its presence is identified as having a higher prevalence in domesticated species compared to wild counterparts, as well as slower-moving species compared to fast and agile mammals (Proks et al. 2018). The morphology of transitional vertebrae is variable, presenting modifications of one or both vertebral arch and body and can have right-to-left asymmetry or lateral cranial-to-caudal gradation in vertebral morphology (Haussler et al. 1997; Breit et al. 2003; Konin and Walz 2010).

In domestic mammals, research on transitional vertebrae is especially focused on dogs and less on cats in which, the congenital abnormalities of the spine are frequently identified radiographically (Bailey and Morgan 1992). The largest description of transitional vertebrae regarding the localisation, the morphology, clinical significance and prevalence exists in dogs (Morgan 1999; Morgan et al. 1999; Damur-Djuric et al. 2006). Such anomalies, as well as others (hemivertebrae, wedge vertebrae, block vertebrae, atlantoaxial malformations and spina bifida) and their prevalence have also been described in cats and ferrets (Newitt et al. 2008; Westworth and Sturges 2010; Proks et al. 2015; Gee Lee et al. 2021). In other domestic mammals, reports regarding the presence and type of transitional vertebrae are even less, for that reason, we proposed to investigate the lumbosacrocaudal spine in as many species as possible, to have a comparative view, not only radiologically, but especially an anatomical analysis. We consider it the first study in which all domestic species are included for this investigation.

Additionally, we intended to highlight the particular morphology of the transitional vertebrae in all the species by anatomical examination, not only radiologically as occurs in most existing research studies on this subject.

## MATERIAL AND METHODS

The research of this study was performed over a period of 14 years, between 2009 and 2023. Studies based on the morphologic description were realised directly on the bones and skeletons in the anatomy labs of The Faculty of Veterinary Medicine from Iasi, Romania, and completed by X-ray exams. In the case of some species – ruminants, horses, pigs, rabbits and cats, the specimens represented old or sick animals with various pathologies (metabolic, locomotor and others), but free of risk for humans. The animals had previously died and the owners agreed to donate them for teaching purposes. After the valorisation of the animals for dissection, their bones were harvested to prepare the skeleton. In this way, over the years, the bones collected were boiled and cleaned of debris mechanically and then chemically in a 3% hydrogen peroxide solution, at variable intervals to obtain clean pieces.

After that, the spine of the animals was analysed anatomically, i.e., the vertebrae were counted and studied for their normal or modified characteristics and described following Nomina Anatomica Veterinaria (NAV) (International Committee on Veterinary Gross Anatomical Nomenclature 2017).

We used 29 cattle specimens, 32 sheep specimens, 31 horse specimens, 26 pig specimens and 33 rabbit specimens. Additionally, the studies involved 89 dogs and 48 cats, from different breeds, both males and females (Table 1). In the case of carnivores, for our purpose, an X-ray exam was chosen as the preferred method, being more facile. The carnivores (as live patients) were the patients of a veterinary clinic where they were subjected to an X-ray exam of the spine.

**Table 1:** Data of the animals of the present research

Species	Total number	Age (years)	Sex (number)		Breed (number)
			♀	♂	
Cattle	29	3–17	16	13	Mixed breed – 19
					Romanian Spotted Cattle – 6
					Romanian Brown – 4
Sheep	32	2–8	19	13	Mixed breed – 20
					Tsigai – 7
					Tsurcana – 5
Horse	31	5–20	24	7	Warmblood horses – 21
					Romanian Saddle Horse – 6
					Romanian Half Heavyweight Horse – 4
Pig	26	2–7	18	8	Hybrids – 19
					Large White Pig – 7
Rabbit	33	1–5	25	8	Mixed breed – 22
					Flemish Giant – 11
Dog	89	6 (months) –12	51	38	Mongrels – 39
					Pekingese – 15
					French Bulldog – 13
					Boxer – 11
					Terrier breeds – 7
					Poodle – 4
Cat	57	1–7	27	30	Common Shorthair – 36
					Scottish Fold – 8
					British Longhair – 7
					Russian Blue – 6

The animals were brought to the clinic for various pathologies, especially presenting nervous and locomotor symptoms. The patients were clinically examined and then the X-ray exam of the spine was performed using latero-lateral and ventro-dorsal projections.

The radiographs obtained were analysed to diagnose the pathology, but were also used for the anatomical conformation of the spine, the number of the vertebrae also being counted. Furthermore, nine common shorthair cats were examined post-mortem, as in the case of ruminants, pigs, horses and rabbits.

The normal vertebral formula on the species was used as reference (Table 2) in order to identify the number variations.

**Table 2:** Normal vertebral formula in domestic mammals

Species	Cervical vertebrae	Thoracic vertebrae	Lumbar vertebrae	Sacral vertebrae	Caudal vertebrae
Cattle	7	13	6	5	18–20
Sheep	7	13	6	4	16–24
Horse	7	18	6 (5)	5	15–21
Pig	7	14–17	6	5 (4)	20–23
Rabbit	7	12	7	4	14–16
Dog	7	13	7	3	20–23
Cat	7	13	7	3	20–23

Source: Ferat-Postolache et al. (2021)

## RESULTS

The investigations performed revealed the presence of transitional and/or supernumerary vertebra in the lumbosacral and sacrococcygeal regions in all the studied species (Table 3).

**Table 3:** The prevalence (%) and type of transitional vertebrae by species

Species	Transitional vertebrae –total number and %	Lumbosacral transitional vertebrae			Sacrocaudal transitional vertebrae	
		sacralisation (L7, L8)	lumbarisation (S1)		sacralisation (Cd1)	caudalisation (last sacral vertebra)
Cattle	3 (8.7%)	–	3		–	–
Sheep	3 (9.37%)	–	2		–	1
Horse	4 (12.9%)	–	–		4	–
Pig	3 (11.53)	–	2		1	–
Rabbit	3* (9.09%)	–	–		–	2
Dog	4* (4.49%)	3	–		–	–
Cat	3 (5.26%)	2	–		–	1

*In the rabbits and dogs, a supernumerary lumbar vertebra that was not sacralised was identified in one specimen

### Ruminants

In the ruminants, we identified transitional vertebrae in both the large and small ruminants (cattle and sheep).

In the cattle, we found the most “spectacular” cases of LTV, more exactly, an evident and intense lumbarisation of the first sacral vertebra (S1). This aspect was found in three animals.

**Figure 1:**
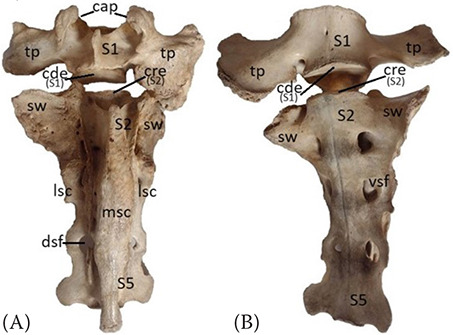
Cattle, female, 9 years – lumbarisation of the first sacral vertebra (S1) (A) Dorsal view. (B) Ventral view cap = cranial articular processes; cde = caudal extremity (*extremitas caudalis*) of S1; cre = cranial extremity (*extremitas cranialis*) of S2; dsf = dorsal sacral foramen; lsc = lateral sacral crest; msc = median sacral crest; S2 = second sacral vertebra; S5 = the fifth (last) sacral vertebra; sw = sacral wing; tp = transverse processes of S1; vsf = ventral sacral foramen

**Figure 2:**
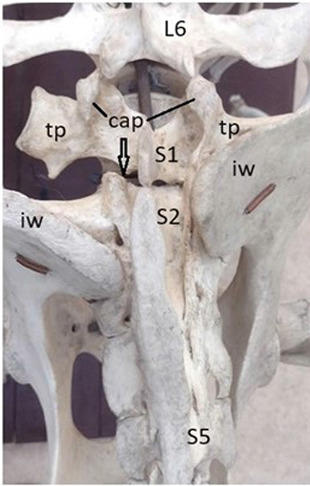
Cattle, male, 4 years – lumbarisation of the first sacral vertebra (S1) and the detachment from the second one (arrow), dorsal view cap = cranial articular processes; iw = iliac wing; L6 = the sixth lumbar vertebra; S2 = second sacral vertebra; S5 = fifth sacral vertebra; tp = transverse processes of S1

In these cases, the first sacral vertebra was independent, having both cranial and caudal extremities on the body. The transverse processes were separated from the wing of the sacrum and tended to be similar to those of the lumbar vertebrae. The two processes were asymmetric in width (Figure 1, Figure 2).

**Figure 3:**
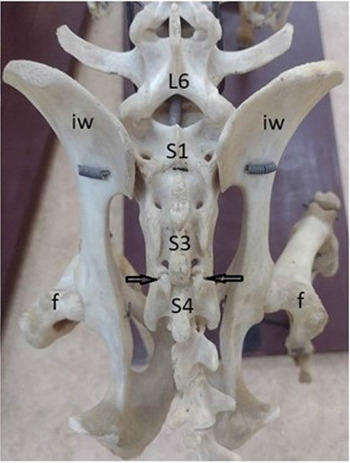
Sheep, male, 4 years – caudalisation of the last sacral vertebra (S4) and detachment from the third one (arrows), dorsal view f = femur; iw = iliac wing; L6 = sixth lumbar vertebra; S1 = first sacral vertebra; S3 = third sacral vertebra

In the sheep, our investigations revealed the presence of transitional vertebrae in the same place as in large ruminants, in two animals, but only a tendency of lumbarisation of the first sacral vertebra was recorded. In another specimen, a sacrocaudal transitional vertebra was found, consisting in caudalisation of the last sacral vertebra that was completely detached (Figure 3).

**Figure 4:**
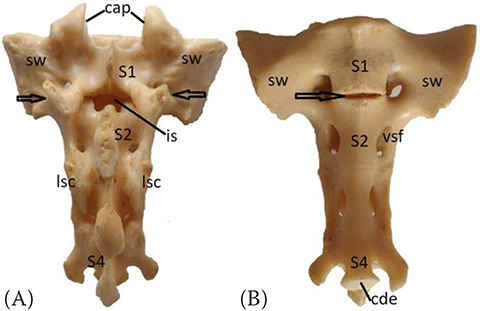
Sheep, female, 3 years – tendency of lumbarisation of the first sacral vertebra (S1); the separation line (arrows) is visible dorsally on the wings between the transverse processes of S1 and S2 together with the presence of the interarcuate space (is) and ventrally by the persistence of intervertebral disc between S1 and S2 (arrow) (A) Dorsal view. (B) Ventral view cap = cranial articular processes; cde = caudal extremity (*extremitas caudalis*) of S4; lsc = lateral sacral crest; S2 = second sacral vertebra; S4 = fourth sacral vertebra; sw = sacral wing; vsf = ventral sacral foramen

In the two cases of lumbarisation, a partial separation of S1 and S2 was observed. Dorsally, in the first case (Figure 4A), the separation was more visible at the level of the arches with a large interarcuate space between the two vertebrae that is normally not present. In the second case, such a space did not exist. In both cases, the tendency of separation was also visible on the sacral wings, where the transverse processes of S1 and S2 were not completely fused (Figure 4A – arrows). Ventrally, on both cases, the incomplete fusion of the vertebral bodies of S1 and S2 and the incomplete ossification of the intervertebral disc (Figure 4B – arrow) could be remarked.

### Horses

**Figure 5:**
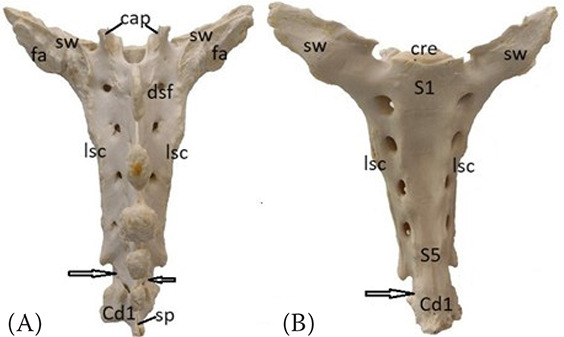
Horse, male, 15 years, warmblood – fusion of the first caudal (Cd1) vertebra and the last sacral vertebra (S5) (A) Dorsal view: ossification process between the arches of Cd1 and S5 and the reduction of the interarcuate space between these vertebrae (arrows). (B) Ventral view: complete fusion of the vertebral bodies of Cd1 and S5 – total ossification of the intervertebral disc (arrow) cap = cranial articular processes; Cd1 = first caudal vertebra; cre = cranial extremity (*extremitas cranialis*) of S1; dsf = dorsal sacral foramen; fa = *facies auricularis*; lsc = lateral sacral crest; S1 = first sacral vertebra; S5 = last sacral vertebra; sp = spinous process of Cd1; sw = sacral wing

Our investigations in this species highlighted the transitional/fused vertebrae in four animals, in the sacrocaudal junction in all the cases. It was noticed that the first caudal vertebra was fused with the last sacral vertebra (S5). As a consequence, the 6 vertabrae accounted for the sacrum bone. In two horses, the vertebral bodies were totally fused due to the complete ossification of the intervertebral disc (Figure 5B). The ossification process was also present between the vertebral arches, and the interarcuate space became narrower (Figure 5A).

**Figure 6:**
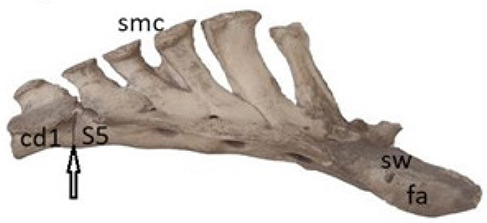
Horse, male, 12 years – the fusion of the first caudal (Cd1) vertebra with the last sacral vertebra (S5), lateral view; the intervertebral disc is ossified, but well visible; no contact between the vertebral arches of Cd1 and S5 cd1 = first caudal vertebra; fa = *facies auricularis*; S5 = last sacral vertebra; smc = sacral median crest (spinous processes); sw = sacral wing

In the other two cases, the fusion happened only between the vertebral bodies, and the intervertebral disc, even if ossified, remained well visible (Figure 6). In all the horses, the transverse processes of the fifth sacral vertebra and the first caudal one remained separated.

### Pigs

In this species, we identified both types of transitional vertebrae, lumbosacral (2 cases) and sacrocaudal (2 cases).

**Figure 7:**
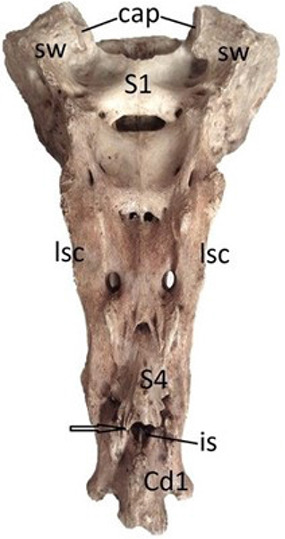
Pig, male, 4 years – the sacralisation of the first caudal vertebra (Cd1), dorsal view: Cd1 is fused with S4 (last sacral vertebra) by their bodies and arches (arrow) and the interarcuate space (is) is narrower cap = cranial articular processes; lsc = lateral sacral crest; S1 = first sacral vertebra; sw = sacral wing

The sacrocaudal transitional vertebra was identified in one pig, consisting in the fusion of the first caudal vertebra with the sacrum bone (sacralisation). The merging occurred both between the bodies and the arches, and secondarily, the interarcuate space became narrower (Figure 7).

**Figure 8:**
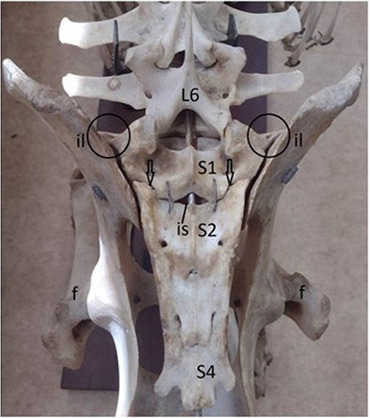
Pig, female, 3 years, dorsal view – lumbarisation of the first sacral vertebra (S1), and detachment from the second one (arrows); tendency of the sacral wings to elongate similar to the transverse processes of the lumbar vertebrae (circles) il = ilium bone; is = interarcuate space; L6 = sixth lumbar vertebra; S2 = second sacral vertebra; S4 = last sacral vertebra

In two other pigs, the detachment of the first sacral vertebra was noticed, i.e., its lumbarisation. The two vertebrae (S1 and S2) were completely separated, but the lumbarisation process was not as intense as in the case of cows, the first sacral vertebra maintaining a more similar conformation as in the case of a normal sacrum bone. Still, a tendency of elongation of the sacral wings to turn into transverse processes could be remarked (Figure 8 – circles).

### Rabbits

**Figure 9:**
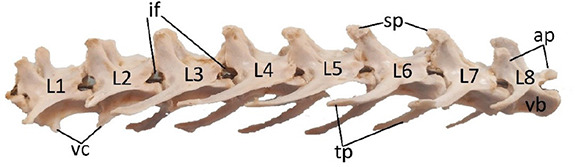
Rabbit, male, 5 years – lumbar spine and the presence of the 8^th^ (L8) lumbar vertebra (supernumerary vertebra – lumbosacral transitional vertebra) ap = articular processes; if = intervertebral foramina; L1–L7 = lumbar vertebrae 1–7; sp = spinous processes; tp = transverse processes; vb = vertebral body; vc = ventral crest

In our examined animals, we identified a supernumerary lumbar vertebra (L8) in a rabbit, the lumbar region counting 8 lumbar vertebrae (Figure 9). The 8^th^ lumbar vertebra had a proper conformation to that of the normal lumbar vertebrae, not fused with adjacent vertebrae. It was only noticed that it was shorter compared with the others. It can be considered as an LTV or a numerical variation of the lumbar vertebrae.

**Figure 10:**
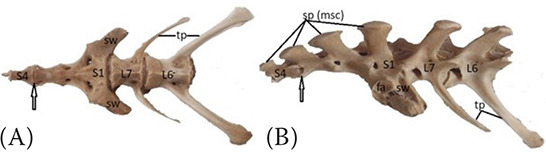
Rabbit, male, 3 years – tendency of caudalisation of the last sacral vertebra (S4) and persistence of the intervertebral disc (arrows) (A) Ventral view. (B) Lateral view fa = *facies auricularis*; L6 = sixth lumbar vertebra; L7 = seventh lumbar vertebra; S1 = first sacral vertebra; S4 = last sacral vertebra; sp (msc) = spinous processes (median sacral crest); sw = sacral wing; tp = transverse processes

In the other two rabbits, the observations revealed the incomplete fusion of the last sacral vertebra with the sacrum bone and the persistence of the intervertebral disc, i.e., a tendency of caudalisation of the last sacral vertebra – S4 (Figure 10).

### Carnivores

**Figure 11:**
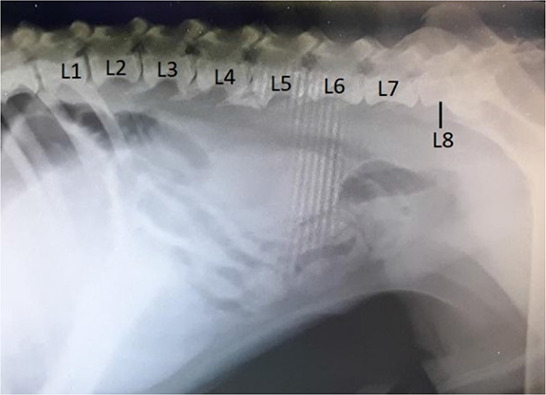
Boxer dog, male, 6 years – supernumerary and transitional lumbosacral vertebra (L8), lateral view: shortening and sacralisation of L8 L1–L7 = lumbar vertebrae 1–7

**Figure 12:**
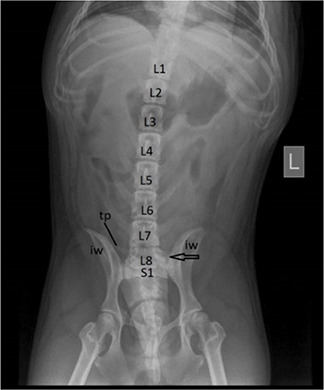
Mongrel dog, female, 14 years – supernumerary and asymmetric transitional lumbosacral vertebra (L8), ventro-dorsal view: shortening and sacralisation of L8 The right transverse process (tp) of L8 is thin and not in contact with the iliac wing (iw), but the left one is fused with that of first sacral vertebra (S1) and makes contact with the iliac wing (arrow) L1–L7 = lumbar vertebrae 1–7

In the dogs, among the various pathologies, radiologically, we identified transitional vertebrae in the lumbosacral junction, having the specific conformation of a lumbar vertebra (supernumerary vertebra) or more or less fused with the sacrum (sacralisation). Three animals had 8 lumbar vertebrae, the last one (L8) being generally shorter (Figure 11, Figure 12), two of them also presenting the sacralisation of L8. In the other dog, the sacralisation of the 7^th^ lumbar vertebra was observed. In all the cases, the sacralisation was partial and asymmetric, one transverse process having a shape resembling that of a normal lumbar transverse process, the other one being fused with the sacral wing and making contact with the iliac wing (Figure 12).

**Figure 13:**
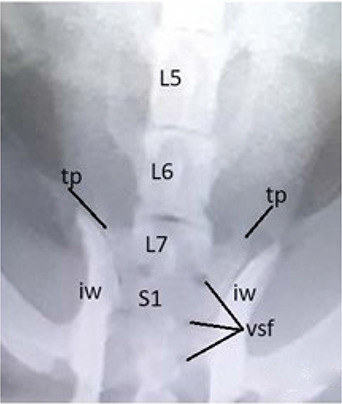
Cat, female, 5 years – transitional lumbosacral vertebra – sacralisation of L7 (last lumbar vertebra), ventro-dorsal view iw = iliac wing; L5 = fifth lumbar vertebra; L6 = sixth lumbar vertebra; S1 = first sacral vertebra; tp = transverse processes of L7 = shortened and transformed into sacral wings; vsf = ventral sacral foramina

**Figure 14:**
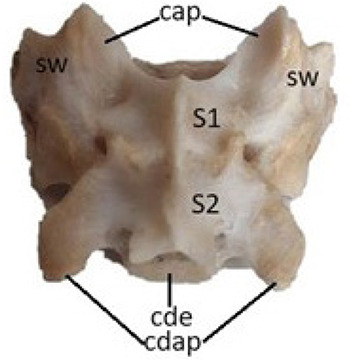
Cat, female, 3 years – 2 sacral vertebrae-caudalisation of the last sacral vertebra (S3), dorsal view cap = cranial articular processes; cdap = caudal articular processes; cde = caudal extremity; S1 = first sacral vertebra; S2 = second sacral vertebra; sw = sacral wing

In the cats, we found transitional vertebrae both in the lumbosacral and sacrocaudal regions. Two animals presented a large degree of sacralisation of the last lumbar vertebra. In one cat, a supernumerary vertebra was found, 8 lumbar vertebrae were counted. The last lumbar vertebra (L8) represented a transitional vertebra, being intensely sacralised. The other cat presented sacralisation of L7. The sacralisation process was similar in both cats, with transverse processes and the vertebral body fused with those of the first sacral vertebra. The last lumbar vertebra (L7 or L8) was observed to be shorter and placed almost entirely between the two iliac wings. There was no evident intervertebral disk between its body and the body of the first sacral vertebra. It was also visible that the transverse processes were shorter cranially and both were fused with those of the first sacral vertebra, being transformed in “wings”. This large fusion resulted in two further large ventral sacral foramina, i.e., on each lateral side of the sacrum, three foramina existed instead of two as in the normal sacrum bone (Ferat-Postolache et al. 2021). The large contact area between the iliac bones and the wings of the sacrum was remarkable, especially on the right side of the pelvis (Figure 13). These aspects were detected during the X-ray examination. Anatomically, in one cat, we identified a sacrum bone counting 2 vertebrae, the last sacral vertebra being independent and caudalised. The second sacral vertebra presented caudal articular processes and caudal extremity of the body (Figure 14).

## DISCUSSION

For ruminants and pigs, very few descriptions of transitional vertebra exist. Occasionally, an unusual transitional vertebra that is difficult to classify is mentioned between the thoracic and lumbar vertebrae in sheep (Pugh et al. 2020). The post-mortem computed tomography (CT) and magnetic resonance image (MRI) scanning of 41 sheep reveals the presence of LTV; this condition is described as an anatomic variation of the last lumbar vertebra in which an enlarged transverse process forms a joint or fusion with the sacrum or ilium. In these sheep, the absence of osteophytes and lesions (such as thinning) of the disc indicate that the fusion is congenital and not due to a degenerative process (Nisolle et al. 2016). In the ruminants, our research revealed LTV, but only with cases of lumbarisation, very evident in case of big ruminants, but not sacralisation.

In pigs, the presence of transitional vertebrae at the thoracolumbar junction is mentioned. In a group of 37 pigs of Landrace and Duroc breeds, the transitional vertebra was identified in 8 pigs (22%), four pigs having 5 lumbar vertebrae and the other four with 6 lumbar vertebrae. Transitional vertebrae only occur at the thoracolumbar junction, representing the thoracoisation of the lumbar vertebrae/lumbar ribs and are counted with thoracic vertebrae. Seven transitional vertebrae were found in the Landrace breed and a single one in the Duroc Breed (Olstad et al. 2022). Thoracolumbar transitional vertebrae are not associated with abnormal curvature in these examined pigs, but in humans, LTV have been associated with lower back pain (Jancuska et al. 2015), and this is worth remembering when considering the relationship between the conformation and spine abnormalities (Olstad et al. 2022). In addition to these observations, we identified transitional vertebrae in pigs, in both lumbosacral and sacrocaudal junction, as the lumbarisation process of the first sacral vertebra and sacralisation of the first caudal vertebra. The variability in the conformation of the spine in pigs, especially in the case of supernumerary vertebrae, can be related to the breeding progress, aiming for a higher number of thoracic and/or lumbar vertebrae, but, in our observations, the two types of transitional vertebrae identified are less probably to be linked with this aspect.

In horses, the data regarding transitional vertebrae are scarce, since it is difficult to realise such as CT scanning or tomography studies on live animals. The only other way is to perform a post-mortem examination. The observations of Haussler et al. (1997), on 36 Thoroughbred racehorses, revealed 6 lumbar and 5 sacral vertebrae that correspond to the “typical” classic vertebral formula in only 61% of the animals. In a high percentage (89%), they report a combined total number of 11 lumbar and sacral vertebrae. The same aspect is highlighted by Stecher (1962) where he also observes that specimens with fewer lumbar vertebrae often have more sacral vertebrae. A reduced number of lumbar vertebrae (5) is also mentioned by other sources (Cotofan et al. 1999; Konig and Liebich 2007; Dyce et al. 2010). Moreover, a higher rate of a reduced number of lumbar vertebrae (5) is reported by Stecher (1962) in donkeys, wild asses and Przewalski’s horses. In donkeys and wild asses, the number of 5 lumbar vertebrae is prevalent, and Przewalski’s horses have 5 and 6 lumbar vertebrae equally divided. Our research in horses did not reveal another number of lumbar vertebrae than 6.

In the same study of Haussler et al. (1997), in eight specimens (22%), thoracolumbar transitional vertebrae are present and in 13 cases (36%), sacrocaudal transitional vertebrae are identified. LTVs are not found, the same as in the case of our research in this species. Other studies reported the sacralisation of L6, characterised by ankyloses and malformation of L6 and the sacrum in two horses with clinical signs of hind limb lameness and possible sacroiliac joint injuries (Jeffcott 1983). Regarding the sacrocaudal vertebrae, it is suggested that the sacrocaudal transitional vertebrae are attributable to congenital development anomalies rather than to acquired ankyloses or pathologic changes (Haussler et al. 1997). Statistically, it has been reported that an elongated sacrum is often seen in aged horses owing to the first and sometimes second caudal vertebra having fused with the sacrum (Haussler et al. 1997). In our findings, only the first caudal vertebra was fused with the sacrum. Similarly, to the study of Haussler et al. (1997), the aspect of “elongated sacrum” was found in our investigations, due to the presence of the sacrocaudal transitional vertebrae in the older specimens, but at a higher rate. Even if this aspect was found in the older animals, we could not determine, with certainty, whether our cases represented an acquired fusion or congenital abnormalities, so we can consider it as transitional vertebrae, as in the study of Haussler et al. (1997). Anyway, anatomically, in both cases, the congenital or acquired abnormality, the result is similar, i.e., the fusion of the two vertebrae.

In the descriptive post-mortem study of Spoor-makers et al. (2021), focused on anatomical variations in three widely differing breeds, the caudal cervical, thoracic, lumbar and sacral regions of the vertebral column of 30 Warmblood horses, 29 Shetland ponies and 18 Konik horses were examined using computed tomography and visualised by volume rendering. Transitional vertebrae were the most frequently encountered in Shetland ponies. Lumbarisation of the last thoracic vertebra was encountered in two (7%) Shetland ponies. In one case, this resulted in a reduction to 17 rib pairs. In the other case, the anomaly resulted in 18 ribs and 7 transverse processes on the left side, and 19 ribs with 6 transverse processes on the right side. The thoracoisation of the first lumbar vertebra was observed in one (3%) Shetland pony, resulting in 19 ribs bilaterally. The sacralisation of the last lumbar vertebra was seen in one (3%) Warmblood. The sacrocaudal transition of the first caudal vertebra (fusion to sacrum) was encountered in 33% of the Shetland ponies, 29% of the Warmblood horses and 6% of the Konik horses. The incomplete sacralisation of the last lumbar vertebra was reported by Scilimati et al. (2023), in a study on the last lumbar vertebrae of 40 horses, by CT scanning. In this case, the sacralisation on the last lumbar vertebra was associated with spondylolisthesis. These results again confirm the higher rate of the sacrocaudal transitional vertebra compared with others in this species (Spoormakers et al. 2021). The development of the imaging techniques led to the possibility of investigating the variability in the conformation and the abnormalities of the spine in this species.

In rabbits, few studies reported the existence of transitional vertebrae located at the junction of different segments of the spine. Malformations caused by transitional vertebrae are essentially observed in the thoracolumbar junction, more rarely at the lumbosacral junction. A thoracolumbar transitional vertebra is associated with lordoscoliosis, wedge vertebrae with kyphoscoliosis, or lordoscoliosis with a twisted pelvis (Chilson et al. 2018). These aspects are confirmed by Proks et al. (2018) in their study, in which the thoracolumbar junction is the most common location of transitional vertebral anomalies and the lumbosacral junction is the second most common location of transitional abnormalities in rabbits. Proks et al. (2018) studied the vertebral formula and the presence of vertebral anomalies in 330 rabbits. Seven vertebral formulas with normal vertebral morphology in 84.8% of animals were identified. The most common abnormalities were the transitional vertebrae found in 41 animals, in the thoracolumbar segment – 35 rabbits, and a lumbosacral one – 6 rabbits. In two rabbits, the presence of thoracolumbar and lumbosacral transitional vertebrae were associated with thoracic lordoscoliosis. No difference between the sexes in the prevalence of anomalies was observed.

Previous investigators suggest that a vertebral formula with eight lumbar vertebrae could increase the susceptibility to lumbar vertebral fractures in rabbits (Gibbs and Hinton 1981); however, we observed this variant in only one rabbit. Few rabbits have a rudimentary intervertebral space between S3 and S4, similar to ferrets (Proks et al. 2015). These findings also correspond with our two cases of the incomplete fusion of the last sacral vertebrae.

The fusion of the last sacral vertebra and the first caudal vertebra has previously been observed in other small animal species, such as cats, dogs and ferrets, and has been described as both a normal morphological variation and as a block vertebra (Newitt et al. 2008; Proks et al. 2015). The presence of five and six sacral vertebrae in rabbits was not described previously (Proks et al. 2015). Congenital vertebral anomalies can cause pain, myelopathy and radiculopathy in small animals (Westworth and Sturges 2010), but most rabbits in the study of Proks et al. (2015) with identifiable congenital vertebral anomalies have no clinical problems associated with the anomalies.

In dogs, the anatomical variations and morphologic abnormalities along the spine, including the transitional vertebrae, have been investigated more often than in other species, due to the possibility of using imaging methods and techniques and the well-known predisposition of this species to these modifications. In dogs, the presence of a supernumerary vertebra (L8) is often reported and usually has no clinical importance (Moeser and Wade 2017). Our observations showed that, in general, the L8 is shorter than the last lumbar vertebra when the lumbar region counts 7 vertebrae. Most problems are reported when the supernumerary vertebra (L8) is associated with LTV. These variations are affected by the sex, developmental factors and breed, with males being more affected than females (Deepa and John 2014). LTV is a common congenital anomaly seen in several dog breeds: Pug, Jack Russell Terrier, French Bulldog, etc. (Lappalainen et al. 2012; Gong et al. 2020). By correlation, our relatively low percentage of transitional vertebrae in dogs can be related to other breeds that have been included in the research and the lower number of animals investigated in this species.

Deepa and John (2014), report that sacralisation is more common than lumbarisation, approximately 2 : 1. In our study, we could also confirm these aspects, transitional vertebrae being found in three males and a single female. No lumbarisation case was found in our group of dogs.

Regarding the shape of the transverse processes (Breit et al. 2003; Damur-Djuric et al. 2006), our investigations revealed only asymmetrical transitional vertebrae.

The presence of LTV predisposes the dogs to develop degenerative lumbosacral stenosis (DLSS) (Ondreka et al. 2013) and can lead to the premature degeneration of the lumbosacral junction and cauda equina syndrome, especially in German Shepherds (Fluckiger et al. 2006; Fluckiger et al. 2009).

In cats, few works have been published relating specifically to the incidence and types of congenital and non-congenital vertebral anomalies. In Manx, spina bifida and hemivertebrae in the caudal and sacral vertebrae are reported (Newitt et al. 2008), but the transitional vertebra is described as the most common abnormality at all levels of the feline spine, the main site for transitional abnormalities being the sacrocaudal junction (Newitt et al. 2008; Newitt et al. 2009). These studies conclude that there is no evidence of any association with clinical signs. In cats, as well as dogs, sacralisation is more prevalent than lumbarisation, all our cases representing strong sacralisation. Harris et al. (2019) observed in their study that LTVs are significantly more prevalent in cats with lumbosacral stenosis compared to the control feline population.

The investigations of Thanaboonnipat et al. (2021) in 1 365 cats revealed the following congenital anomalies: six lumbar vertebrae (141), sacralisation (48), lumbarisation (16), hemivertebrae (1), and five lumbar vertebrae (1). Even if the number of animals is higher, the percentage of LTV (sacralisation + lumbarisation) in their study corresponds to those of our findings. Thanaboonnipat et al. (2021) concluded that cats with abnormal lumbosacral vertebrae are prone to having more problems with the large bowel. Moreover, congenital and acquired lumbosacral abnormalities are often accompanied by the risk of large bowel abnormalities (Thanaboonnipat et al. 2021).

In all the investigated species, we could not establish a direct relation between the presence of transitional vertebrae and other criteria, especially with the breed, since most of the animals were of mixed breed. Regarding age, many cases were observed in relatively old animals, but we can affirm this, at most, in the case of the sacralisation of Cd1, where we consider it a result of the ossification process due to age, rather than a congenital malformation. Further investigations might lead to more precise data. Regarding sex, it is difficult to affirm the predisposition of males or females to transitional vertebrae. Most of the species under study included a higher number of males, but transitional vertebrae were found both in males and females, except in the dogs, where the transitional vertebrae occurred predominantly in males.

In conclusion, even if the majority of the research on transitional vertebrae has been focused on carnivores, especially on dogs, we can affirm that it occurs in most domestic mammals, at least in the lumbosacral and sacrocaudal junctions. We also found a large morphological variability in the transitional vertebrae among the investigated species. In carnivores, in accordance with other research, it is obvious that the sacralisation process and supernumerary vertebrae predominate. An evident lumbarisation process occurred in our study, mostly in ruminants, both big and small, but also in pigs, these findings being among the first in this direction. The transitional sacrocaudal vertebrae seem to appear especially in horses, then in pigs as a more or less intense ossification process in the case of old animals. In rabbits, transitional vertebrae also occur in both the lumbosacral and sacrocaudal junctions, even if the cases in our findings were not very spectacular.
